# Genetic parameters for body weight and different definitions of residual feed intake in broiler chickens

**DOI:** 10.1186/s12711-019-0494-2

**Published:** 2019-09-23

**Authors:** Wossenie Mebratie, Per Madsen, Rachel Hawken, Hélène Romé, Danye Marois, John Henshall, Henk Bovenhuis, Just Jensen

**Affiliations:** 1 Center for Quantitative Genetics and Genomics, Department of Molecular Biology and Genetics, Aarhus University, 8830 Tjele, Denmark; 2 Animal Breeding and Genomics Centre, Wageningen University, P.O. Box 338, 6700 AH Wageningen, The Netherlands; 3 Cobb-Vantress Inc., Siloam Springs, AR 72761-1030 USA; 4 College of Agriculture and environmental sciences, Bahir Dar University, P.O. Box 5501, Bahir Dar, Ethiopia

## Abstract

**Background:**

The objectives of this study were to (1) simultaneously estimate genetic parameters for BW, feed intake (FI), and body weight gain (Gain) during a FI test in broiler chickens using multi-trait Bayesian analysis; (2) derive phenotypic and genetic residual feed intake (RFI) and estimate genetic parameters of the resulting traits; and (3) compute a Bayesian measure of direct and correlated superiority of a group selected on phenotypic or genetic residual feed intake. A total of 56,649 male and female broiler chickens were measured at one of two ages (${\text{t}}$ or ${\text{t}} - 6$ days). BW, FI, and Gain of males and females at the two ages were considered as separate traits, resulting in a 12-trait model. Phenotypic RFI (${\text{RFI}}_{\text{P}}$) and genetic RFI (${\text{RFI}}_{\text{G}}$) were estimated from a conditional distribution of FI given BW and Gain using partial phenotypic and partial genetic regression coefficients, respectively.

**Results:**

Posterior means of heritability for BW, FI and Gain were moderately high and estimates were significantly different between males and females at the same age for all traits. In addition, the genetic correlations between male and female traits at the same age were significantly different from 1, which suggests a sex-by-genotype interaction. Genetic correlations between ${\text{RFI}}_{\text{P}}$ and ${\text{RFI}}_{\text{G }}$ were significantly different from 1 at an older age but not at a younger age.

**Conclusions:**

The results of the multivariate Bayesian analyses in this study showed that genetic evaluation for production and feed efficiency traits should take sex and age differences into account to increase accuracy of selection and genetic gain. Moreover, for communicating with stakeholders, it is easier to explain results from selection on ${\text{RFI}}_{\text{G}}$ than selection on ${\text{RFI}}_{\text{P}}$, since ${\text{RFI}}_{\text{G}}$ is genetically independent of production traits and it explains the efficiency of birds in nutrient utilization independently of energy requirements for production and maintenance.

## Background

Genetic improvement of body weight (BW) and feed efficiency (FE) traits has received major consideration in the poultry industry due to their economic and environmental implications. Body weight is the live weight of birds at a given age, while feed efficiency is the ability of birds to convert kg of feed into kg of body weight gain. Among the different ways of measuring FE in poultry, residual feed intake (RFI) is a popular partial measure of FE due to its phenotypic independence of production traits, the presence of considerable variation in RFI among birds and the moderately high heritability of the trait [1]. Classically, RFI is defined as the difference between actual feed intake and predicted feed intake based on energy requirements for production (e.g. body weight, body weight gain) and maintenance [2]. Since RFI is phenotypically independent of production traits, variation in RFI reflects differences in efficiency with which birds use feed for production and maintenance of BW, as well as errors in its prediction [3].

Prediction of RFI has been largely based on phenotypic regression of feed intake on measures of production from multiple regression analysis. This is called phenotypic RFI since direct consideration has not been given to underlying genetic regressions in the computation of RFI and it is not genetically independent of production traits. Kennedy et al. [3] derived phenotypic RFI using phenotypic regression coefficients, which were obtained from phenotypic co(variance) matrices of feed intake and production traits that were assumed to be known without error, and showed that phenotypic RFI is phenotypically independent of production traits but not genetically independent of production traits. In the same context, Kennedy et al. [3] proposed the term genetic RFI to explain the component of feed intake that is genetically independent of production traits and derived by using genetic regression coefficients obtained from genetic co(variance) matrices of feed intake and production traits that were assumed to be known without error. In this regard, direct consideration is not given to phenotypic regressions in the computation of genetic RFI (${\text{RFI}}_{\text{G}}$), therefore, ${\text{RFI}}_{\text{G}}$ is not phenotypically independent of production traits [3].

Jensen et al. [4] extended the definitions of phenotypic and genetic RFI provided by Kennedy et al. [3] to a Bayesian framework, which estimates the co(variance) function for RFI, using proper distributions of feed intake conditional on production traits in a one-step procedure. This one-step procedure estimates the partial phenotypic and genetic regression coefficients from co(variance) matrices of feed intake and production traits that result from a multiple trait analysis of feed intake and production traits, and then simultaneously derives phenotypic and genetic RFI within the model. This one-step approach properly accounts for errors in the estimation of regression coefficients as compared to the classical two-step approach of Kennedy et al. [3], which assumes co(variance) matrices of component traits of RFI to be known without error and that of Koch et al. [2]. In the classical two-step procedures, first feed intake and production traits are analyzed in a multiple regression analysis and regression coefficients are obtained before genetic analysis. Then, RFI for each animal is computed using the regression coefficients and the genetic analysis is performed together with production or body composition traits. In these procedures, the resulting covariance functions of RFI and production traits are usually singular because RFI is defined before genetic analysis as a linear combination of other traits [4]. However, the one-step procedure in the Bayesian analysis avoids singularity of the co(variance) matrices by simultaneously estimating the co(variance) functions of RFI based on the conditional distribution of FI given production and possibly body composition traits [4]. In addition, the Bayesian one-step procedure ensures that parameter estimation in the regression analysis is not biased by including “fixed effects” in the model and effects due to genetic trends for component traits in the population under study [4].

Thus, the objectives of this study were to (1) simultaneously estimate genetic parameters for body weight, feed intake, and body weight gain in broiler chickens from a multi-trait Bayesian analysis; (2) derive phenotypic and genetic RFI and estimate genetic parameters for these traits; and (3) compute a Bayesian measure of direct and correlated superiority of a group selected on phenotypic or genetic residual feed intake.

## Methods

### Data

Data from 16 selection rounds (SR) for growth rate of broiler chickens, which were reared under strict bio-secure environmental conditions, were collected using procedures by Cobb-Vantress. In total, 56,649, male and female broilers with phenotypic data were available. For the first 10 SRs, body weight was recorded at ${\text{t}}$ days of age in males $\left( {{\text{BW}}_{{{\text{m }}\left( {\text{t}} \right)}} } \right)$ and females $\left( {{\text{BW}}_{{{\text{f}}\left( {\text{t}} \right)}} } \right)$, however, as selection continued, the birds started to mature earlier and the age at which they were weighed was changed to 6 days earlier (${\text{t}} - 6$ days) for the last six SRs in males $\left( {{\text{BW}}_{{{\text{m }}\left( {{\text{t}} - 6} \right)}} } \right)$ and females $\left( {{\text{BW}}_{{{\text{f }}\left( {{\text{t}} - 6} \right)}} } \right)$. The heaviest 22,281 male and female birds in the two periods (${\text{t}}$ and ${\text{t}} - 6$ days of age) entered to the feed efficiency (FE) test, and feed intake (FI) was recorded over the test period on males (${\text{FI}}_{{{\text{m}}\left( {\text{t }} \right)}}$) and females (${\text{FI}}_{{{\text{f }}\left( {\text{t }} \right)}}$) at ${\text{t}}$ days of age or on males $\left( {{\text{FI}}_{{{\text{m}}\left( {{\text{t}} - 6} \right)}} } \right)$ and females $\left( {{\text{FI}}_{{{\text{f }}\left( {{\text{t}} - 6} \right)}} } \right)$ at ${\text{t}} - 6$ days of age. The proportion of birds that entered the FE test was the same for each selection round. Body weight gain during the FE test for males $\left( {{\text{Gain}}_{{{\text{m }}\left( {\text{t }} \right)}} } \right)$ and females $\left( {{\text{Gain}}_{{{\text{f}}\left( {\text{t}} \right) }} } \right)$ at ${\text{t}}$ days of age or body weight gain (Gain) for males $\left( {{\text{Gain}}_{{{\text{m }}\left( {{\text{t}} - 6} \right)}} } \right)$ and females $\left( {{\text{Gain}}_{{{\text{f }}\left( {{\text{t}} - 6} \right) }} } \right)$ at ${\text{t}} - 6$ days of age were calculated as the difference between final body weight and body weight at the start of the FE test.

### Statistical model and estimation of parameters

Preliminary univariate and bivariate restricted maximum likelihood estimation (REML) analyses were conducted to determine whether BW, FI and Gain in males and females at the two ages should be considered as the same trait or separate traits. The REML results showed that genetic parameters were significantly different between sexes (males and females) and at the two ages (${\text{t}}$ and ${\text{t}} - 6$ days), which suggest that records on males and females as well as records at both ages should be considered as separate traits in the subsequent analyses. This resulted in four different traits for each of the BW, FI, and Gain traits. For example, BW for males at two different periods with different recording ages $\left( {{\text{BW}}_{{{\text{m }}\left( {\text{t}} \right)}} } \right)$ and ${\text{BW}}_{{{\text{m}}\left( {{\text{t}} - 6} \right)}}$ and BW for females at two different periods with different recording ages $\left( {{\text{BW}}_{{{\text{f}}\left( {\text{t}} \right)}} \;{\text{and}}\;{\text{BW}}_{{{\text{f}}\left( {{\text{t}} - 6} \right)}} } \right)$. Thus, the final analysis was a Bayesian multivariate (12-trait) analysis using Gibbs sampling where the following model was specified for each of the BW, FI and Gain traits in males and females and the two ages (${\text{t}}$ and ${\text{t}} - 6$ days).

1 $${\mathbf{y}}_{{{\mathbf{ijkl}}}} = {\text{SRH}}_{\text{j}} + {\text{Dage}}_{\text{k}} + {\text{a}}_{\text{i}} + {\text{pe}}_{\text{l}} + {\text{e}}_{\text{ijkl}} ,$$

where ${\mathbf{y}}_{{{\mathbf{ijkl}}}}$ is a vector of ${\text{BW}}_{{{\text{m}}\left( {\text{t}} \right)}}$, ${\text{BW}}_{{{\text{f}}\left( {\text{t}} \right)}}$, ${\text{BW}}_{{{\text{m}}\left( {{\text{t}} - 6} \right)}}$, ${\text{BW}}_{{{\text{f}}\left( {{\text{t}} - 6} \right)}}$, ${\text{FI}}_{{{\text{m}}\left( {\text{t}} \right)}}$, ${\text{FI}}_{{{\text{f}}\left( {\text{t}} \right)}}$, ${\text{FI}}_{{{\text{m}}\left( {{\text{t}} - 6} \right)}}$, ${\text{FI}}_{{{\text{f}}\left( {{\text{t}} - 6} \right)}}$, ${\text{Gain}}_{{{\text{m}}\left( {\text{t}} \right)}}$, ${\text{Gain}}_{{{\text{f}}\left( {\text{t}} \right)}}$, ${\text{Gain}}_{{{\text{m}}\left( {{\text{t}} - 6} \right)}}$, and ${\text{Gain}}_{{{\text{f}}\left( {{\text{t}} - 6} \right)}}$ observations of chicken ${\text{i}}$ that hatched in the selection round and hatch batch ${\text{j}}$, ${\text{SRH}}_{\text{j}}$ is the interaction between selection round of chicken ${\text{i}}$ and hatch batch ${\text{j}}$ of the individual chicken, ${\text{Dage}}_{\text{k}}$ is the effect of ${\text{k}}^{\text{th}}$ age of dam, ${\text{k}}$ having 32 levels, ${\text{a}}_{\text{i}}$ is the additive genetic effect of chicken ${\text{i}}$, ${\text{pe}}_{\text{l}}$ is the maternal permanent environmental effect of dam ${\text{l}}$, and, ${\text{e}}_{\text{ijkl}}$ is the random residual effect of chicken ${\text{i}}$. Flat priors were used for all “fixed” location and dispersion parameters. Prior distributions for random vectors in the model were: ${\mathbf{a}}\sim {\text{N}}\left( {0,{\mathbf{A}} \otimes {\mathbf{G}}_{0} } \right)$, ${\mathbf{pe}}\sim {\text{N}}\left( {0,{\mathbf{I}} \otimes {\mathbf{K}}_{0} } \right)$ and ${\mathbf{e}}\sim {\text{N}}\left( {0, {\mathbf{I}} \otimes {\mathbf{R}}_{0} } \right)$, where ${\mathbf{A}}$ is the numerator relationship matrix, ${\mathbf{G}}_{0}$ is the co (variance) matrix for direct additive genetic effects of dimension 12 (four variance components for each of the BW, FI and Gain traits), ${\mathbf{I}}$ is an identity matrix, ${\mathbf{K}}_{0}$ is the co (variance) matrix for maternal permanent environmental effect of dimension 12, and ${\mathbf{R}}_{0}$ is the residual co (variance) matrix of dimension 12, which was assumed to be heterogeneous with a different variance for each of the 12 traits. Symbol $\otimes$ denotes the Kronecker (direct) product. The random effects ${\mathbf{a}}$, ${\mathbf{pe}}$, and ${\mathbf{e}}$ were considered independent of each other. The Gibbs sampler was used to obtain posterior distributions for all parameters included in the 12-trait model. The Gibbs sampler was run for 1,000,000 rounds; the first 250,000 rounds were discarded as burn-in and from the remaining samples, every 250th sample was saved for post-Gibbs analysis. The RJMC module in the DMU software package was used for analysis [5].

### Posterior distributions of parameters

From the joint posterior distribution of all location and (co) variance parameters of the 12-trait model, 3000 samples were saved for post-Gibbs analysis. Posterior means of breeding values and co (variance) components were computed as the average of samples after the burn-in period. The boa package in the R program was used to evaluate convergence of co (variance) parameters [6]. Functions to define the posterior distribution of genetic, maternal permanent environment and residual (co)variances for the 12 original traits ${\text{BW}}_{{{\text{m}}\left( {\text{t}} \right)}}$, ${\text{BW}}_{{{\text{f}}\left( {\text{t}} \right)}}$, ${\text{BW}}_{{{\text{m}}\left( {{\text{t}} - 6} \right)}}$, ${\text{BW}}_{{{\text{f}}\left( {{\text{t}} - 6} \right)}}$, ${\text{FI}}_{{{\text{m}}\left( {\text{t}} \right)}}$, ${\text{FI}}_{{{\text{f}}\left( {\text{t}} \right)}}$, ${\text{FI}}_{{{\text{m}}\left( {{\text{t}} - 6} \right)}}$, ${\text{FI}}_{{{\text{f}}\left( {{\text{t}} - 6} \right)}}$, ${\text{Gain}}_{{{\text{m}}\left( {\text{t}} \right)}}$, ${\text{Gain}}_{{{\text{f}}\left( {\text{t}} \right)}}$, ${\text{Gain}}_{{{\text{m}}\left( {{\text{t}} - 6} \right)}}$, ${\text{Gain}}_{{{\text{f}}\left( {{\text{t}} - 6} \right)}}$ and the eight derived RFI traits namely, phenotypic RFI for males at ${\text{t}}$ days of age $\left( {{\text{RFI}}_{{{\text{P}}\left( {\text{m tdays}} \right)}} } \right),$ phenotypic RFI for females at ${\text{t}}$ days of age $\left( {{\text{RFI}}_{{{\text{P}}\left( {\text{f tdays}} \right)}} } \right),$ phenotypic RFI for males at ${\text{t}} - 6$ days of age $\left( {{\text{RFI}}_{{{\text{P}}\left( {{\text{m t}} - 6{\text{days}}} \right)}} } \right),$ phenotypic RFI for females at ${\text{t}} - 6$ days of age $\left( {{\text{RFI}}_{{{\text{P}}\left( {{\text{f t}} - 6{\text{days}}} \right)}} } \right)$, genetic RFI for males at ${\text{t}}$ days of age $\left( {{\text{RFI}}_{{{\text{G}}\left( {\text{m tdays}} \right)}} } \right)$, genetic RFI for females at ${\text{t}}$ days of age $\left( {{\text{RFI}}_{{{\text{G}}\left( {\text{f tdays}} \right)}} } \right)$, genetic RFI for males at ${\text{t}} - 6$ days of age $\left( {{\text{RFI}}_{{{\text{G}}\left( {{\text{m t}} - 6{\text{days}}} \right)}} } \right)$, and genetic RFI for females at ${\text{t}} - 6$ days of age $\left( {{\text{RFI}}_{{{\text{G}}\left( {{\text{f t}} - 6{\text{days}}} \right)}} } \right)$ were derived based on Shirali et al. [7]. Phenotypic RFI (${\text{RFI}}_{\text{P}}$) and genetic RFI (${\text{RFI}}_{\text{G}}$) were derived using phenotypic partial regression coefficients and genetic partial regression coefficients, respectively for each sex and age class. This ensures that the phenotypic covariance between ${\text{RFI}}_{\text{P}}$ and production traits (BW and Gain) is zero and the genetic covariance between ${\text{RFI}}_{\text{G}}$ and production traits is zero, since ${\text{RFI}}_{\text{P}}$ and ${\text{RFI}}_{\text{G}}$ are linear combinations of BW, FI and Gain. For ${\text{RFI}}_{\text{P}}$, the partial phenotypic regression coefficients (${\mathbf{b}}_{\text{p}}$) for BW and Gain were computed from the phenotypic (co)variance matrix while for ${\text{RFI}}_{\text{G}}$, the partial genetic regression coefficients (${\mathbf{b}}_{\text{g}}$) for BW and Gain were computed from the genetic (co)variance matrix. Within a posterior sample, the two RFI definitions have conditional normal distributions, and were derived as follows: let ${\mathbf{P}}_{0} = {\mathbf{G}}_{0} + {\mathbf{K}}_{0} + {\mathbf{R}}_{0}$ be the phenotypic and ${\mathbf{G}}_{0}$ the genetic (co)variance matrices, which were computed based on each sample from the posterior distribution of parameters, where ${\mathbf{P}}_{0}$ is 12 × 12 phenotypic co(variance) matrix, ${\mathbf{K}}_{0}$ is 12 × 12 maternal permanent environmental co(variance) matrix, ${\mathbf{R}}_{0}$ is 12 × 12 residual co (variance) matrix. Bayesian estimates of partial phenotypic (${\mathbf{b}}_{\text{P}}$) and genetic (${\mathbf{b}}_{\text{G}}$) regression coefficients were computed as follows:

2 $$\begin{aligned} & {\mathbf{b}}_{\text{P}} = {\mathbf{P}}_{{\mathbf{P}}}^{ - 1} {\mathbf{P}}_{{{\text{P}},{\text{FI}}}} , \\ & {\text{and}}\; {\mathbf{b}}_{\text{G}} = {\mathbf{G}}_{{\mathbf{P}}}^{ - 1} {\mathbf{G}}_{{{\text{P}},{\text{FI}}}} , \\ \end{aligned}$$

where ${\mathbf{b}}_{\text{P}}$ and ${\mathbf{b}}_{\text{G}}$ are 2 × 1 vectors obtained in each sample from the Gibbs output for each sex and age; ${\mathbf{P}}_{{\mathbf{P}}}$ and ${\mathbf{G}}_{\text{P}}$ are 2 × 2 phenotypic and genetic (co)variance matrices for the production traits of BW and Gain from ${\mathbf{P}}_{0 }$ and ${\mathbf{G}}_{0}$, respectively for each sex and age; and ${\mathbf{P}}_{{{\text{P}},{\text{FI}}}}$ and ${\mathbf{G}}_{{{\text{P}},{\text{FI}}}}$ are phenotypic and genetic (co)variance matrices, respectively, between the production traits (BW and Gain) and FI in each sex and age. The predicted breeding values for ${\text{RFI}}_{\text{P}}$ can be obtained for all animals from the conditional distribution of breeding values for FI, given the breeding values of BW and Gain, using ${\mathbf{b}}_{\text{P}}$, and the predicted breeding values for ${\text{RFI}}_{\text{G}}$ can be obtained for all animals from the conditional distribution of breeding values for FI, given the breeding values of BW and Gain using ${\mathbf{b}}_{\text{G}}$ [7]. For example, a given sample from the posterior distribution of breeding values for phenotypic (${\mathbf{a}}_{\text{RFIP}}$) and genetic (${\mathbf{a}}_{\text{RFIG}}$) RFI can be computed as follows:

3 $${\mathbf{a}}_{\text{RFIP}} = {\mathbf{a}}_{\text{FI}} - \left[ {{\mathbf{a}}_{\text{Gain}} {\mathbf{a}}_{\text{BW}} } \right]{\mathbf{b}}_{\text{P}} ,$$

4 $${\mathbf{a}}_{\text{RFIG}} = {\mathbf{a}}_{\text{FI }} - \left[ {{\mathbf{a}}_{\text{Gain}} {\mathbf{a}}_{\text{BW}} } \right]{\mathbf{b}}_{\text{G}} .$$

The distribution of ${\text{RFI}}_{\text{P}}$ for a given sample was obtained as the distribution of FI, conditional on BW and Gain. Similarly, the distribution of ${\text{RFI}}_{\text{G}}$ for a given sample was obtained as the distribution of FI, conditional on BW and Gain. The corresponding co(variances) can be computed as follows:

5 $$\begin{aligned} & {\mathbf{P}}_{1} = {\mathbf{B}}*{\mathbf{P}}_{0} \varvec{*}{\mathbf{B^{\prime}}}, \\ & {\text{and}}\;{\mathbf{G}}_{1} = {\mathbf{B}}*{\mathbf{G}}_{0} * {\mathbf{B^{\prime}}}, \\ \end{aligned}$$

where ${\mathbf{P}}_{1}$ is the new 20 × 20 phenotypic co (variance) matrix and ${\mathbf{G}}_{1}$ is the new 20 × 20 genetic co (variance) matrix that includes the eight derived traits of RFI, in addition to the original 12 traits included in the Bayesian multivariate model. ${\mathbf{P}}_{0 } \varvec{ }$ and ${\mathbf{G}}_{0}$ are 12 × 12 phenotypic and genetic co (variance) matrices of the original traits, respectively and, ${\mathbf{B}}$ represents 20 linear functions used to derive the new set of traits. The first 12 are identity functions for the 12 original traits and the remaining eight are functions for the eight derived traits of RFI. Details are in Shirali et al. [7].

### Superiority of the selected group

The Bayesian measure of the direct and correlated genetic superiority of a group of selected birds was calculated as the difference between the mean of the predicted breeding values in the selected population and the mean of the population corrected for genetic trend. Unlike the classical selection index calculations, it is possible to make probability statements on the expected response to selection in the Bayesian analysis and their posterior standard deviations can be derived easily. This gives an expression of superiority of the selected group in each sample from the posterior distribution. The mean of the selected group for trait ${\text{j}}$ when selecting on trait ${{\text{j}^{\prime}}}$ was calculated based on the formula following Shirali et al. [7] as:

6 $${\text{a}}_{{\text{jj}^{\prime}}}^{{\text{s}}} = \frac{1}{{{\text{n}}_{{\text{s}}} }}\sum\limits_{{{\text{i}} = 1}}^{{\text{n}}} {{\text{a}}_{{{\text{ij}}}} } {\mathbf{I}}({\text{a}}_{{\text{ij}^{\prime}}} > {\text{a}}_{{{\text{n}}_{{\text{s}}} \text{j}^{\prime}}} ),$$

where ${\text{a}}_{{{{\text{jj}^{\prime}}}}}^{\text{s}}$ is the mean of the selected group for trait ${\text{j}}$ when selection is on trait ${{\text{j}^{\prime}}}$, ${\text{a}}_{\text{ij}}$ is the sampled additive breeding value for trait ${\text{j}}$ on animal ${\text{i}}$ conditional on the genetic trend; ${\text{n}}$ is the total number of animals, and ${\text{a}}_{{{\text{n}}_{\text{s}} {{\text{j}^{\prime}}}}}$ is the sampled additive genetic value for a ranked individual (${\text{n}}_{\text{s}}$) when ordering animals based on sampled breeding values for trait ${{\text{j}^{\prime}}}$. If ${\text{j}} = {{\text{j}^{\prime}}}$, the superiority is for the trait under selection, and if ${\text{j}} \ne {{\text{j}^{\prime}}}$, the superiority is for trait ${\text{j}}$ due to selection of a possibly correlated trait ${{\text{j}^{\prime}}}$. This study includes 20 traits (12 original and 8 derived traits). Therefore, 20 scenarios were developed to compare direct and correlated superiorities of the selected group for production or feed efficiency traits. However, only the results of the eight scenarios for the derived traits are presented. The number of individuals ranked for the analysis was decided based on truncation selection of the best 5, 10 and 20% of animals, however, only the results of truncation selection when selecting the best 10% are presented, because the results are consistent across all selection percentages. Production traits (BW and Gain), were selected upwards and FE traits (FI, ${\text{RFI}}_{\text{P}}$ and ${\text{RFI}}_{\text{G}}$) were selected downwards.

## Results

### Genetic parameters of production and feed efficiency traits

Descriptive statistics of the data are in Table 1. Males had a higher mean BW, FI and Gain than females at the two ages. The higher mean BW in males than females is consistent with previous studies [8, 9].

**Table 1:** Descriptive statistics of the data

Trait	N	Mean (g)	SD	Min	Max
${\text{BW}}_{{{\text{m}}\left( {\text{t}} \right)}}$	17,270	2732.3	268.6	1776.0	3712.0
${\text{BW}}_{{{\text{f}}\left( {\text{t}} \right)}}$	18,461	2303.3	221.9	1463.0	3045.0
${\text{BW}}_{{{\text{m}}\left( {{\text{t}} - 6} \right)}}$	10,117	2182.8	213.3	1319.0	2890.0
${\text{BW}}_{{{\text{f}}\left( {{\text{t}} - 6} \right)}}$	10,801	1882.4	180.2	1077.0	2449.0
${\text{FI}}_{{{\text{m}}\left( {\text{t}} \right)}}$	5065	2851.7	215.6	1919.0	3712.0
${\text{FI}}_{{{\text{f}}\left( {\text{t}} \right)}}$	10,334	2355.1	185.2	1700.0	3045.0
${\text{FI}}_{{{\text{m}}\left( {{\text{t}} - 6} \right)}}$	2301	2311.3	163.1	1493.0	2890.0
${\text{FI}}_{{{\text{f}}\left( {{\text{t}} - 6} \right)}}$	4581	1954.3	141.3	1421.0	2449.0
${\text{Gain}}_{{{\text{m}}\left( {\text{t}} \right)}}$	5056	619.2	146.6	206.0	1161.0
${\text{Gain}}_{{{\text{f}}\left( {\text{t}} \right)}}$	10,239	479.3	102.6	182.0	865.0
${\text{Gain}}_{{{\text{m}}\left( {{\text{t}} - 6} \right)}}$	2299	636.4	115.4	292.0	960.0
${\text{Gain}}_{{{\text{f}}\left( {{\text{t}} - 6} \right)}}$	4533	469.2	91.3	211.0	725.0

$BW_{m\left( t \right)}$ body weight of males at ${\text{t}}$ days of age, $BW_{f\left( t \right)}$ body weight of females at ${\text{t}}$ days of age, $BW_{{m\left( {t - 6} \right)}}$ body weight of males at ${\text{t}} - 6$ days of age, $BW_{{f\left( {t - 6} \right)}}$ body weight of females at ${\text{t}} - 6$ days of age, $FI_{m\left( t \right)}$ feed intake of males at ${\text{t}}$ days of age, $FI_{f\left( t \right)}$ feed intake of females at ${\text{t}}$ days of age, $FI_{{m\left( {t - 6} \right)}}$ feed intake of males at ${\text{t}} - 6$ days of age, $FI_{{f\left( {t - 6} \right)}}$ feed intake of females at ${\text{t}} - 6$ days of age, $Gain_{m\left( t \right)}$ body weight gain of males at ${\text{t}}$ days of age, $Gain_{f\left( t \right)}$ body weight gain of females at ${\text{t}}$ days of age, $Gain_{{m\left( {t - 6} \right)}}$ body weight gain of males at ${\text{t}} - 6$ days of age, $Gain_{{f\left( {t - 6} \right)}}$ body weight gain of females at ${\text{t}} - 6$ days of age, *RFI* residual feed intake

The posterior mean and posterior standard deviations (PSD) of genetic variance, residual variance, and heritability for the 12 original traits and the eight derived traits of RFI are in Table 2, in which all trait abbreviations are also summarized. All the reported parameters are posterior means, which were computed as averages of 3000 samples from the posterior distribution. For ease of presentation and interpretation of the results, comparison of genetic parameters for males and females is limited to the same age, and comparison of genetic parameters for the two ages is limited to the same sex in “Results” and “Discussion” sections. The genetic variance of BW for males and females was significantly different, males having a larger genetic variance than females at both ages. However, heritability estimates were higher for females than males at both ages. Similarly, the genetic variance of BW was significantly different at both ages and decreased as the weighing age decreased from ${\text{t}}$ to ${\text{t}} - 6$ days. However, the heritability estimates of BW increased slightly as weighing age decreased due to the relatively higher residual variance at the older age than the younger age.

**Table 2:** Posterior means of variance components and heritability of body weight (BW), feed intake (FI), body weight gain (Gain), and two RFI traits (${\text{RFI}}_{\text{P}}$ and ${\text{RFI}}_{\text{G}}$), with their posterior standard deviations (PSD)

Traits	$\sigma_{\text{a}}^{ 2}$ (g^2^)	PSD	$\sigma_{\text{e}}^{ 2}$ (g^2^)	PSD	$\sigma_{\text{pe}}^{ 2}$ (g^2^)	PSD	${\text{h}}^{ 2}$	PSD
${\text{BW}}_{{{\text{m}}\left( {\text{t}} \right)}}$	17,865.5	1870.0	37,592.8	1056.0	1868.5	391.4	0.31	0.03
${\text{BW}}_{{{\text{f}}\left( {\text{t}} \right)}}$	13,339.2	1349.4	25,141.9	747.1	1519.5	282.4	0.33	0.03
${\text{BW}}_{{{\text{m}}\left( {{\text{t}} - 6} \right)}}$	12,277.1	1643.8	23,589.6	902.1	1578.6	354.2	0.33	0.04
${\text{BW}}_{{{\text{f}}\left( {{\text{t}} - 6} \right)}}$	10,228.5	1263.3	15,983.8	680.3	1116.8	249.8	0.37	0.04
${\text{FI}}_{{{\text{m}}\left( {\text{t}} \right)}}$	8014.2	993.4	17,993.3	647.0	976.5	280.0	0.30	0.03
${\text{FI}}_{{{\text{f}}\left( {\text{t}} \right)}}$	4926.2	535.9	13,493.1	339.9	616.3	140.6	0.26	0.03
${\text{FI}}_{{{\text{m}}\left( {{\text{t}} - 6} \right)}}$	6981.7	1178.8	10,445.3	706.1	971.7	312.9	0.38	0.05
${\text{FI}}_{{{\text{f}}\left( {{\text{t}} - 6} \right)}}$	4645.4	568.7	8343.7	350.6	447.1	144.0	0.35	0.04
${\text{Gain}}_{{{\text{m}}\left( {\text{t}} \right)}}$	4053.8	576.9	13,212.1	414.5	549.6	174.1	0.23	0.03
${\text{Gain}}_{{{\text{f}}\left( {\text{t}} \right)}}$	1664.5	210.4	6841.4	147.9	231.8	58.3	0.19	0.02
${\text{Gain}}_{{{\text{m}}\left( {{\text{t}} - 6} \right)}}$	4307.4	798.3	7686.0	490.6	607.0	203.2	0.34	0.05
${\text{Gain}}_{{{\text{f}}\left( {{\text{t}} - 6} \right)}}$	2099.1	297.3	5124.7	195.6	239.9	76.7	0.28	0.04
${\text{RFI}}_{{{\text{P}}\left( {\text{m tdays}} \right)}}$	1431.2	247.1	6260.3	183.8	290.7	83.7	0.18	0.03
${\text{RFI}}_{{{\text{P}}\left( {\text{f tdays}} \right)}}$	961.1	143.0	4878.0	103.0	231.1	48.6	0.16	0.02
${\text{RFI}}_{{{\text{P}}\left( {{\text{m t}} - 6{\text{days}}} \right)}}$	1443.0	295.7	3369.7	191.4	272.0	89.3	0.28	0.05
${\text{RFI}}_{{{\text{P}}\left( {{\text{f t}} - 6{\text{days}}} \right)}}$	747.0	128.3	2243.2	83.0	117.8	35.4	0.24	0.04
${\text{RFI}}_{{{\text{G}}\left( {\text{m tdays}} \right)}}$	1261.0	246.1	7171.2	537.7	299.5	90.7	0.14	0.03
${\text{RFI}}_{{{\text{G}}\left( {\text{f tdays}} \right)}}$	874.8	145.7	5241.3	207.1	256.9	54.3	0.14	0.02
${\text{RFI}}_{{{\text{G}}\left( {{\text{m t}} - 6{\text{days}}} \right)}}$	1380.8	297.5	3660.2	365.5	287.9	96.9	0.26	0.05
${\text{RFI}}_{{{\text{G}}\left( {{\text{f t}} - 6{\text{days}}} \right)}}$	720.5	128.0	2357.2	134.0	128.3	40.6	0.22	0.04

$BW_{m\left( t \right)}$ body weight of males at ${\text{t}}$ days of age, $BW_{f\left( t \right)}$ body weight of females at ${\text{t}}$ days of age, $BW_{{m\left( {t - 6} \right)}}$ body weight of males at ${\text{t}} - 6$ days of age, $BW_{{f\left( {t - 6} \right)}}$ body weight of females at ${\text{t}} - 6$ days of age, $FI_{m\left( t \right)}$ feed intake of males at ${\text{t}}$ days of age, $FI_{f\left( t \right)}$ feed intake of females at ${\text{t}}$ days of age, $FI_{{m\left( {t - 6} \right)}}$ feed intake of males at ${\text{t}} - 6$ days of age, $FI_{{f\left( {t - 6} \right)}}$ feed intake of females at ${\text{t}} - 6$ days of age, $Gain_{m\left( t \right)}$ body weight gain of males at ${\text{t}}$ days of age, $Gain_{f\left( t \right)}$ body weight gain of females at ${\text{t}}$ days of age, $Gain_{{m\left( {t - 6} \right)}}$ body weight gain of males at ${\text{t}} - 6$ days of age, $Gain_{{f\left( {t - 6} \right)}}$ body weight gain of females at ${\text{t}} - 6$ days of age, *RFI* residual feed intake, $RFI_{{P\left( {m tdays } \right)}}$ phenotypic RFI of males at ${\text{t}}$ days of age, $RFI_{{P\left( {f tdays} \right)}}$ phenotypic RFI of females at ${\text{t}}$ days of age, $RFI_{{P\left( {m t - 6days} \right)}}$ phenotypic RFI of males at ${\text{t}} - 6$ days of age, $RFI_{{P\left( {f t - 6days} \right)}}$ phenotypic RFI of females at ${\text{t}} - 6$ days of age, $RFI_{{G\left( {m tdays} \right)}}$ genetic RFI of males at ${\text{t}}$ days of age, $RFI_{{G\left( {f tdays} \right)}}$ genetic RFI of females at ${\text{t}}$ days of age, $RFI_{{G\left( {m t - 6days} \right)}}$ genetic RFI of males at ${\text{t}} - 6$ days of age, $RFI_{{G\left( {f t - 6 days} \right)}}$ genetic RFI of females at ${\text{t}} - 6$ days of age, *g* gram, *PSD* posterior standard deviation

Unlike BW traits, the heritability estimates of FI, Gain, ${\text{RFI}}_{\text{P}}$ and ${\text{RFI}}_{\text{G}}$ were higher for males than females at both ages except for ${\text{RFI}}_{\text{G}}$ at ${\text{t}}$ days of age for which heritability estimates were the same for males and females. The posterior means of the heritability estimates of FI and Gain were low to moderate (0.19–0.38) for males and females at both ages, with males having significantly higher estimates than females. Moreover, the genetic variance and heritability estimates of FI were significantly higher than that of Gain for males and females at both ages (Table 2). The heritability estimates of the two RFI definitions were low (0.14–0.28); with ${\text{RFI}}_{\text{P}}$ having slightly higher heritability estimates than ${\text{RFI}}_{\text{G}}$ for both sexes and at both ages. This was followed by a slightly higher genetic variance for ${\text{RFI}}_{\text{P}}$ than ${\text{RFI}}_{\text{G}}$ and higher residual variance for ${\text{RFI}}_{\text{G}}$ than ${\text{RFI}}_{\text{P}}$ for both sexes and at both ages.

The posterior means of the genetic correlations between the 12 traits included in the multivariate model are in Table 3. PSD of correlations are not shown to simplify presentation of results, however, they were within a lower range (0.01–0.06). The posterior mean of the genetic correlation of BW (PSD in parentheses) between males and females at ${\text{t}}$ days was 0.90 (0.02) and the genetic correlation of BW between males and females at ${\text{t}} - 6$ days was 0.90 (0.03). The genetic correlations of FI between males and females were 0.82 (0.04) and 0.86 (0.04) at ${\text{t}}$ and ${\text{t}} - 6$ days of age, respectively. The genetic correlations of Gain between males and females were 0.75 (0.06) and 0.81 (0.06) at ${\text{t}}$ and ${\text{t}} - 6$ days of age, respectively with genetic correlations being significantly higher at early ages (${\text{t}} - 6$ days) than later ages (${\text{t}}$) for both FI and Gain.

**Table 3:** Posterior means of genetic correlations between body weight (BW), feed intake (FI), and body weight gain (Gain)

Trait	${\text{BW}}_{{{\text{m}}\left( {\text{t}} \right)}}$	${\text{BW}}_{{{\text{f}}\left( {\text{t}} \right)}}$	${\text{BW}}_{{{\text{m}}\left( {t - 6} \right)}}$	${\text{BW}}_{{{\text{f}}\left( {{\text{t}} - 6} \right)}}$	${\text{FI}}_{{{\text{m}}\left( {\text{t}} \right)}}$	${\text{FI}}_{{{\text{f}}\left( {\text{t}} \right)}}$	${\text{FI}}_{{{\text{m}}\left( {{\text{t}} - 6} \right)}}$	${\text{FI}}_{{{\text{f}}\left( {{\text{t}} - 6} \right)}}$	${\text{Gain}}_{{{\text{m}}\left( {\text{t}} \right)}}$	${\text{Gain}}_{{{\text{f}}\left( {\text{t}} \right)}}$	${\text{Gain}}_{{{\text{m}}\left( {{\text{t}} - 6} \right)}}$
$${\text{BW}}_{{{\text{m}}\left( {\text{t}} \right)}}$$											
${\text{BW}}_{{{\text{f}}\left( {\text{t}} \right)}}$	0.90^a^										
${\text{BW}}_{{{\text{m}}\left( {{\text{t}} - 6} \right)}}$	0.84^g^	0.82									
${\text{BW}}_{{{\text{f}}\left( {{\text{t}} - 6} \right)}}$	0.78	0.87^g^	0.90^b^								
${\text{FI}}_{{{\text{m}}\left( {\text{t}} \right)}}$	*0.49*	0.41	0.44	0.37							
${\text{FI}}_{{{\text{f}}\left( {\text{t}} \right)}}$	0.53	*0.60*	0.52	0.59	0.82^c^						
${\text{FI}}_{{{\text{m}}\left( {{\text{t}} - 6} \right)}}$	0.50	0.44	*0.51*	0.48	0.76^g^	0.75					
${\text{FI}}_{{{\text{f}}\left( {{\text{t}} - 6} \right)}}$	0.60	0.57	0.55	*0.63*	0.77	0.88^g^	0.86^d^				
${\text{Gain}}_{{{\text{m}}\left( {\text{t}} \right)}}$	*0.25*	0.20	0.25	0.16	*0.87*	0.64	0.59	0.55			
${\text{Gain}}_{{{\text{f}}\left( {\text{t}} \right)}}$	0.21	*0.30*	0.24	0.29	0.69	*0.82*	0.60	0.68	0.75^e^		
${\text{Gain}}_{{{\text{m}}\left( {{\text{t}} - 6} \right)}}$	0.46	0.42	*0.45*	0.42	0.67	0.68	*0.88*	0.73	0.64^g^	0.68	
${\text{Gain}}_{{{\text{f}}\left( {{\text{t}} - 6} \right)}}$	0.49	0.47	0.47	*0.50*	0.73	0.79	0.82	*0.89*	0.67	0.78^g^	0.81^f^

Trait descriptions are as defined in Table 2

Numbers in italic characters are genetic correlations between traits at the same sex and age

^a–f^Genetic correlations of traits between males and females within ages

^g^Genetic correlations of traits between ages within sexes

Posterior means of genetic correlations between BW and FI were moderate (0.49–0.63) for males and females at both ages. Genetic correlations between BW and Gain were also moderate (0.30–0.50) for males and females at both ages, except for the low genetic correlation (0.25) between BW and Gain for males at ${\text{t}}$ days of age. Genetic correlations between FI and Gain were high (0.82–0.90) for males and females at both ages (Table 3). Posterior means of genetic correlations between the two RFI definitions are in Table 4. Genetic correlations between ${\text{RFI}}_{\text{P}}$ and ${\text{RFI}}_{\text{G}}$ at the same sex and age were high and significantly different from 1 at ${\text{t}}$ days of age, however, at ${\text{t}} - 6$ days of age, genetic correlations were not significantly different from 1 (Table 4).

**Table 4:** Posterior means of genetic correlations (PSD in parentheses) between phenotypic and genetic residual feed intake (${\text{RFI}}_{\text{P}}$ and ${\text{RFI}}_{\text{G}} ,$ respectively)

Trait	${\text{RFI}}_{{{\text{P}}\left( {\text{m tdays}} \right)}}$	${\text{RFI}}_{{{\text{P}}\left( {\text{f tdays}} \right)}}$	${\text{RFI}}_{{{\text{P}}\left( {{\text{m t}} - 6 \,{\text{days}}} \right)}}$	${\text{RFI}}_{{{\text{P}}\left( {{\text{f t}} - 6 \,{\text{days}}} \right)}}$	${\text{RFI}}_{{{\text{G}}\left( {\text{m tdays}} \right)}}$	${\text{RFI}}_{{{\text{G}}\left( {\text{f tdays}} \right)}}$	${\text{RFI}}_{{{\text{G}}\left( {{\text{m t}} - 6\, {\text{days}}} \right)}}$
$${\text{RFI}}_{{{\text{P}}\left( {\text{m tdays}} \right)}}$$							
${\text{RFI}}_{{{\text{P}}\left( {\text{f tdays}} \right)}}$	0.80 (0.06)^a^						
${\text{RFI}}_{{{\text{P}}\left( {{\text{m t}} - 6{\text{days}}} \right)}}$	0.68 (0.11)^b^	0.63 (0.14)					
${\text{RFI}}_{{{\text{P}}\left( {{\text{f t}} - 6{\text{days}}} \right)}}$	0.70 (0.10)	0.71 (0.09)^b^	0.70 (0.09)^a^				
${\text{RFI}}_{{{\text{G}}\left( {\text{m tdays}} \right)}}$	*0.94 (0.04)*	0.77 (0.07)	0.67 (0.12)	0.72 (0.10)			
${\text{RFI}}_{{{\text{G}}\left( {\text{f tdays}} \right)}}$	0.78 (0.07)	*0.95 (0.02)*	0.64 (0.16)	0.71 (0.10)	0.80 (0.07)^a^		
${\text{RFI}}_{{{\text{G}}\left( {{\text{m t}} - 6{\text{days}}} \right)}}$	0.65 (0.11)	0.60 (0.15)	*0.98 (0.02)*	0.69 (0.09)	0.66 (0.12)^b^	0.63 (0.16)	
${\text{RFI}}_{{{\text{G}}\left( {{\text{f t}} - 6{\text{days}}} \right)}}$	0.67 (0.10)	0.67 (0.10)	0.67 (0.09)	*0.98 (0.02* **)**	0.71 (0.11)	0.69 (0.11)^b^	0.68 (0.09)^a^

Abbreviations are as defined in Table 2

Numbers in italic characters are genetic correlations between ${\text{RFI}}_{\text{P}}$ and ${\text{RFI}}_{\text{G}}$ at the same sex and age

^a^Genetic correlations of ${\text{RFI}}_{\text{P}}$ and ${\text{RFI}}_{\text{G}}$ between sexes within ages

^b^Genetic correlations of ${\text{RFI}}_{\text{P}}$ and ${\text{RFI}}_{\text{G}}$ between ages within sexes

Genetic and phenotypic correlations between production traits and the two RFI definitions are in Table 5. ${\text{RFI}}_{\text{G}}$ was derived using genetic partial regression coefficients of FI on production traits. Therefore, the posterior mean of genetic correlations between ${\text{RFI}}_{\text{G}}$ and BW at the same sex and age were equal to 0 (results not shown) and the genetic correlations between ${\text{RFI}}_{\text{P}}$ and BW at the same sex and age were low (0.04–0.24) but different from 0 (Table 5). Similarly, ${\text{RFI}}_{\text{P}}$ was derived using phenotypic partial regression coefficients of FI on production traits. Therefore, phenotypic correlations between ${\text{RFI}}_{\text{P}}$ and BW at the same sex and age were equal to 0 (results not shown) and phenotypic correlations between ${\text{RFI}}_{\text{G}}$ and BW at the same sex and age were low (− 0.002 to − 0.15) but different from 0 (Table 5).

**Table 5:** Posterior means of genetic and phenotypic correlations (PSD in parentheses) of production traits (BW and Gain) with two RFI traits (${\text{RFI}}_{\text{P}}$ and ${\text{RFI}}_{\text{G}}$)

Production traits	${\text{RFI}}_{\text{P}}$	Genetic correlation	Production traits	${\text{RFI}}_{\text{G}}$	Phenotypic correlation
${\text{BW}}_{{{\text{m}}\left( {\text{t}} \right)}}$	${\text{RFI}}_{{{\text{P}}\left( {\text{m tdays}} \right)}}$	0.09 (0.09)	${\text{BW}}_{{{\text{m}}\left( {\text{t}} \right)}}$	${\text{RFI}}_{{{\text{G}}\left( {\text{m tdays}} \right)}}$	− 0.04 (0.07)
${\text{BW}}_{{{\text{f}}\left( {\text{t}} \right)}}$	${\text{RFI}}_{{{\text{P}}\left( {\text{f tdays}} \right)}}$	0.24 (0.08)	${\text{BW}}_{{{\text{f}}\left( {\text{t}} \right)}}$	${\text{RFI}}_{{{\text{G}}\left( {\text{f tdays}} \right)}}$	− 0.15 (0.06)
${\text{BW}}_{{{\text{m}}\left( {{\text{t}} - 6} \right)}}$	${\text{RFI}}_{{{\text{P}}\left( {{\text{m t}} - 6{\text{days}}} \right)}}$	0.04 (0.11)	${\text{BW}}_{{{\text{m}}\left( {{\text{t}} - 6} \right)}}$	${\text{RFI}}_{{{\text{G}}\left( {{\text{m t}} - 6{\text{days}}} \right)}}$	− 0.002 (0.12)
${\text{BW}}_{{{\text{f}}\left( {{\text{t}} - 6} \right)}}$	${\text{RFI}}_{{{\text{P}}\left( {{\text{f t}} - 6{\text{days }}} \right)}}$	0.10 (0.09)	${\text{BW}}_{{{\text{f}}\left( {{\text{t}} - 6} \right)}}$	${\text{RFI}}_{{{\text{G}}\left( {{\text{f t}} - 6{\text{days }}} \right)}}$	− 0.05 (0.08)
${\text{Gain}}_{{{\text{m}}\left( {\text{t}} \right)}}$	${\text{RFI}}_{{{\text{P}}\left( {\text{m tdays}} \right)}}$	0.32 (0.10)	${\text{Gain}}_{{{\text{m}}\left( {\text{t}} \right)}}$	${\text{RFI}}_{{{\text{G}}\left( {\text{m tdays}} \right)}}$	− 0.27 (0.08)
${\text{Gain}}_{{{\text{f}}\left( {\text{t}} \right)}}$	${\text{RFI}}_{{{\text{P}}\left( {\text{f tdays}} \right)}}$	0.20 (0.09)	${\text{Gain}}_{{{\text{f}}\left( {\text{t}} \right)}}$	${\text{RFI}}_{{{\text{G}}\left( {\text{f tdays}} \right)}}$	− 0.14 (0.08)
${\text{Gain}}_{{{\text{m}}\left( {{\text{t}} - 6} \right)}}$	${\text{RFI}}_{{{\text{P}}\left( {{\text{m t}} - 6{\text{days }}} \right)}}$	0.11 (0.13)	${\text{Gain}}_{{{\text{m}}\left( {{\text{t}} - 6} \right)}}$	${\text{RFI}}_{{{\text{G}}\left( {{\text{m t}} - 6{\text{days }}} \right)}}$	− 0.10 (0.13)
${\text{Gain}}_{{{\text{f}}\left( {{\text{t}} - 6} \right)}}$	${\text{RFI}}_{{{\text{P}}\left( {{\text{f t}} - 6{\text{days }}} \right)}}$	0.12 (0.10)	${\text{Gain}}_{{{\text{f}}\left( {{\text{t}} - 6} \right)}}$	${\text{RFI}}_{{{\text{G}}\left( {{\text{f t}} - 6{\text{days }}} \right)}}$	− 0.09 (0.10)

Abbreviations are as defined in Table 2

The same holds true for genetic and phenotypic correlations between the two RFI definitions and Gain. Posterior means of genetic correlations between ${\text{RFI}}_{\text{G}}$ and Gain at the same sex and age were confirmed to be 0 (results not shown) and genetic correlations between ${\text{RFI}}_{\text{P}}$ and Gain at the same sex and age were low to moderate (0.11–0.32) (Table 5). Similarly, phenotypic correlations between ${\text{RFI}}_{\text{P}}$ and Gain at the same sex and age were confirmed to be 0 (results not shown) and phenotypic correlations between ${\text{RFI}}_{\text{G}}$ and Gain at the same sex and age were low (− 0.09 to − 0.27) but different from 0 (Table 5).

Estimates of genetic and phenotypic correlations between FI and the two RFI definitions are in Table 6. The correlations were moderate for both males and females and at the two ages, with higher genetic correlations between FI and ${\text{RFI}}_{\text{P}}$ (0.51–0.66) than between FI and ${\text{RFI}}_{\text{G}}$ (0.39–0.45). Phenotypic correlations between FI and ${\text{RFI}}_{\text{P}}$ at the same sex and age ranged from 0.48 to 0.57, and phenotypic correlations between FI and ${\text{RFI}}_{\text{G}}$ at the same sex and age ranged from 0.31 to 0.43, but were lower than the genetic correlations between FI and the two RFI definitions (Table 6).

**Table 6:** Posterior means of genetic and phenotypic correlations (PSD in parentheses) of feed intake with two RFI traits (${\text{RFI}}_{\text{P}}$ and ${\text{RFI}}_{\text{G}}$)

Feed intake	Two RFI definitions	Genetic correlation	Phenotypic correlation
${\text{FI}}_{{{\text{m}}\left( {\text{t}} \right)}}$	${\text{RFI}}_{{{\text{P}}\left( {\text{m tdays}} \right)}}$	0.66 (0.06)	0.54 (0.01)
${\text{FI}}_{{{\text{f}}\left( {\text{t}} \right)}}$	${\text{RFI}}_{{{\text{P}}\left( {\text{f tdays}} \right)}}$	0.64 (0.05)	0.57 (0.01)
${\text{FI}}_{{{\text{m}}\left( {{\text{t}} - 6} \right)}}$	${\text{RFI}}_{{{\text{P}}\left( {{\text{m t}} - 6{\text{days}}} \right)}}$	0.54 (0.09)	0.53 (0.01)
${\text{FI}}_{{{\text{f}}\left( {{\text{t}} - 6} \right)}}$	${\text{RFI}}_{{{\text{P}}\left( {{\text{f t}} - 6{\text{days }}} \right)}}$	0.51 (0.07)	0.48 (0.01)
${\text{FI}}_{{{\text{m}}\left( {\text{t}} \right)}}$	${\text{RFI}}_{{{\text{G}}\left( {\text{m tdays}} \right)}}$	0.40 (0.04)	0.31 (0.08)
${\text{FI}}_{{{\text{f}}\left( {\text{t}} \right)}}$	${\text{RFI}}_{{{\text{G}}\left( {\text{f tdays}} \right)}}$	0.42 (0.04)	0.41 (0.06)
${\text{FI}}_{{{\text{m}}\left( {{\text{t}} - 6} \right)}}$	${\text{RFI}}_{{{\text{G}}\left( {{\text{m t}} - 6{\text{days }}} \right)}}$	0.45 (0.06)	0.43 (0.11)
${\text{FI}}_{{{\text{f}}\left( {{\text{t}} - 6} \right)}}$	${\text{RFI}}_{{{\text{G}}\left( {{\text{f t}} - 6{\text{days }}} \right)}}$	0.39 (0.04)	0.39 (0.08)

Abbreviations are as defined in Table 2

### Superiority of the selected group

Table 7 shows the posterior mean of the direct and correlated genetic level of the selected groups under single trait selection for low ${\text{RFI}}_{\text{P}}$ and ${\text{RFI}}_{\text{G}}$. Direct selection for low ${\text{RFI}}_{\text{P}}$ in males at ${\text{t}}$ days of age resulted in a direct selection response of − 63.0 g and a correlated response of decreasing ${\text{RFI}}_{\text{G}}$ by 55.6 g at the same age for males. Similarly, direct selection for low ${\text{RFI}}_{\text{G}}$ in males at ${\text{t}}$ days of age resulted in a direct selection response of − 59.4 g and a correlated response of decreasing ${\text{RFI}}_{\text{P}}$ by 59.0 g at the same age in males. As shown in Table 7, the correlated response in ${\text{RFI}}_{\text{G}}$ from direct selection on ${\text{RFI}}_{\text{P}}$ was slightly different at ${\text{t}}$ days of age, however, the correlated response in ${\text{RFI}}_{\text{P}}$ was the same as the direct response on ${\text{RFI}}_{\text{G}}$ at both ages. The slight differences in the correlated response at ${\text{t}}$ days of age might be because the genetic correlations between ${\text{RFI}}_{\text{P}}$ and ${\text{RFI}}_{\text{G}}$ at ${\text{t}}$ days of were significantly different from 1.

**Table 7:** Posterior means of direct (numbers in italic characters) and correlated (non-italic characters in a row) additive genetic superiority when the best 10% were selected based on low ${\text{RFI}}_{\text{P}}$ or ${\text{RFI}}_{\text{G}}$

Trait	${\text{RFI}}_{{{\text{P}}\left( {\text{m t}} \right)}}$	${\text{RFI}}_{{{\text{P}}\left( {\text{f t}} \right)}}$	${\text{RFI}}_{{{\text{P}}\left( {{\text{m t}} - 6} \right)}}$	${\text{RFI}}_{{{\text{P}}\left( {{\text{f t}} - 6} \right)}}$	${\text{RFI}}_{{{\text{G}}\left( {\text{m t}} \right)}}$	${\text{RFI}}_{{{\text{G}}\left( {\text{f t}} \right)}}$	${\text{RFI}}_{{{\text{G}}\left( {{\text{m t}} - 6} \right)}}$	${\text{RFI}}_{{{\text{G}}\left( {{\text{f t}} - 6} \right)}}$
${\text{RFI}}_{{{\text{P}}\left( {\text{m tdays}} \right)}}$	− *63.0*	− 40.7	− 42.5	− 31.8	− 55.6	− 37.8	− 39.8	− 29.8
${\text{RFI}}_{{{\text{P}}\left( {\text{f tdays}} \right)}}$	− 49.7	− *51.7*	− 39.0	− 32.5	− 45.2	− 47.2	− 36.7	− 30.0
${\text{RFI}}_{{{\text{P}}\left( {{\text{m t}} - 6{\text{days}}} \right)}}$	− 42.0	− 31.7	− *63.5*	− 31.5	− 38.8	− 30.7	− 60.8	− 29.8
${\text{RFI}}_{{{\text{P}}\left( {{\text{f t}} - 6{\text{days}}} \right)}}$	− 43.5	− 36.5	− 43.6	− *45.9*	− 42.2	− 34.5	− 42.4	− 44.4
${\text{RFI}}_{{{\text{G}}\left( {\text{m t days}} \right)}}$	− 59.0	− 39.4	− 41.6	− 32.8	− *59.4*	− 39.2	− 40.5	− 31.8
${\text{RFI}}_{{{\text{G}}\left( {\text{f t days}} \right)}}$	− 48.3	− 49.4	− 39.7	− 32.2	− 47.1	− *49.4*	− 38.5	− 31.1
${\text{RFI}}_{{{\text{G}}\left( {{\text{m t}} - 6{\text{days}}} \right)}}$	− 40.2	− 30.4	− 62.0	− 31.3	− 38.5	− 30.3	− *62.2*	− 30.2
${\text{RFI}}_{{{\text{G}}\left( {{\text{f t}} - 6{\text{days}}} \right)}}$	− 41.3	− 34.2	− 41.8	− 45.0	− 41.5	− 33.8	− 41.5	− *45.3*

Abbreviations are as defined in Table 2

Table 8 shows the posterior means of the genetic level of the selected groups for correlated traits under single trait selection for low ${\text{RFI}}_{\text{P}}$ or ${\text{RFI}}_{\text{G}}$. Direct selection for low ${\text{RFI}}_{\text{P}}$ at ${\text{t}}$ days of age in males had a correlated response of decreasing FI by 98.2 g, while decreasing BW and Gain by 17.3 and 33.8 g, respectively at ${\text{t}}$ days of age in males. Thus, direct selection for low ${\text{RFI}}_{\text{P}}$ had a favorable correlated response of decreasing FI by a relatively larger amount than decreasing BW and Gain. Moreover, direct selection for low ${\text{RFI}}_{\text{G}}$ in males at ${\text{t}}$ days of age resulted in a correlated response of decreasing FI by 58.2 g at the same age in males, with no significant change in BW and Gain. Similarly, direct selection for low ${\text{RFI}}_{\text{G}}$ in females at ${\text{t}} - 6$ days decreased FI by − 43.2 g, with no significant change on BW and Gain at ${\text{t}} - 6$ days of age in females (Table 8). As expected from the definition, direct selection on ${\text{RFI}}_{\text{G}}$ did not show considerable correlated response on BW and Gain at the same sex and age, however, it resulted in a correlated response of decreasing FI (Table 8).

**Table 8:** Posterior means of correlated (numbers in a row) additive genetic superiority of the selected group when the best 10% were selected based on low ${\text{RFI}}_{\text{P}}$ or ${\text{RFI}}_{\text{G}}$

Trait	${\text{BW}}_{{{\text{m}}\left( {\text{t}} \right)}}$	${\text{BW}}_{{{\text{f}}\left( {\text{t}} \right)}}$	${\text{BW}}_{{{\text{m}}\left( {{\text{t}} - 6} \right)}}$	${\text{BW}}_{{{\text{f}}\left( {{\text{t}} - 6} \right)}}$	${\text{FI}}_{{{\text{m}}\left( {\text{t}} \right)}}$	${\text{FI}}_{{{\text{f}}\left( {\text{t}} \right)}}$	${\text{FI}}_{{{\text{m}}\left( {{\text{t}} - 6} \right)}}$	${\text{FI}}_{{{\text{f}}\left( {{\text{t}} - 6} \right)}}$	${\text{Gain}}_{{{\text{m}}\left( {\text{t}} \right)}}$	${\text{Gain}}_{{{\text{f}}\left( {\text{t}} \right)}}$	${\text{Gain}}_{{{\text{m}}\left( {{\text{t}} - 6} \right)}}$	${\text{Gain}}_{{{\text{f}}\left( {{\text{t}} - 6} \right)}}$
${\text{RFI}}_{{{\text{P}}\left( {\text{m tdays}} \right)}}$	− *17.3*	− 5.2	− 12.9	− 15.7	− *98.2*	− 65.9	− 71.6	− 62.2	− *33.8*	− 21.8	− 28.1	− 25.7
${\text{RFI}}_{{{\text{P}}\left( {\text{f tdays}} \right)}}$	− 63.7	− *47.6*	− 50.1	− 54.5	− 79.6	− *74.4*	− 69.7	− 67.7	− 19.0	− *12.5*	− 25.6	− 24.8
${\text{RFI}}_{{{\text{P}}\left( {{\text{m t}} - 6{\text{days}}} \right)}}$	− 10.8	1.1	− *6.4*	− 10.6	− 58.0	− 39.4	− *76.3*	− 56.2	− 14.8	− 7.2	− *12.1*	− 21.1
${\text{RFI}}_{{{\text{P}}\left( {{\text{f t}} - 6{\text{days}}} \right)}}$	− 38.1	− 20.0	− 3.6	− *17.8*	− 54.3	− 46.9	− 51.1	− *56.2*	− 3.8	− 6.0	− 7.2	− *7.0*
${\text{RFI}}_{{{\text{G}}\left( {\text{m tdays}} \right)}}$	− *0.4*	6.90	0.52	− 8.8	− *58.2*	− 43.6	− 46.2	− 43.6	*1.0*	− 4.9	− 4.6	− 8.7
${\text{RFI}}_{{{\text{G}}\left( {\text{f tdays}} \right)}}$	− 20.6	− *3.3*	− 16.5	− 21.0	− 56.1	− *49.0*	− 48.6	− 46.6	− 4.1	*0.8*	− 7.1	− 10.3
${\text{RFI}}_{{{\text{G}}\left( {{\text{m t}} - 6{\text{days}}} \right)}}$	− 3.1	6.9	− *0.5*	− 5.8	− 47.4	− 31.0	− *62.3*	− 47.4	− 7.0	− 1.7	− *0.3*	− 13.9
${\text{RFI}}_{{{\text{G}}\left( {{\text{f t}} - 6{\text{days}}} \right)}}$	− 20.8	− 4.5	11.6	− 2.7	− 42.0	− 35.1	− 37.0	− *43.2*	3.5	− 0.03	3.6	*2.0*

Abbreviations are as defined in Table 2

Numbers in italic characters are correlated responses within sexes and ages

## Discussion

### Genetic parameters of production and feed efficiency traits

We used a Bayesian method to analyze genetic parameters for production and feed efficiency traits for male and female broiler chickens recorded at two different ages, to derive phenotypic and genetic RFI, and to estimate a Bayesian measure of direct and correlated superiority of a group of animals selected on feed efficiency traits. The Bayesian method allows posterior means of the two RFI definitions to be derived simultaneously within the model, with their posterior standard deviations, while integrating over all unknown model parameters, including “fixed” and dispersion parameters.

#### Genetic parameters for body weight, feed intake, and body weight gain

Different authors have estimated genetic parameters for BW of males and females at different ages of broiler chickens and reported heritability estimates ranging from 0.20 to 0.53 [8, 10–12]. Our heritability estimates of BW for males and females were moderate (0.31–0.37) at both ages and within the range of those in previous studies. Moreover, our results showed that the genetic variance of BW was higher for males than females at both ages. The higher genetic variance for males than females might be partly due to a scale effect because males had a higher mean BW than females at a given age; however, the heritability estimates were significantly higher for females than for males due to higher residual variance for males. The genetic correlation of BW between males and females was significantly different from 1 at both ages, which is in agreement with results of Mebratie et al. [8]. The difference in heritability estimates between BW for males and females, and the lower than 1 genetic correlations of BW between males and females suggest that there is sex-by-genotype interaction for BW in broiler chickens. This implies that, genes may react differently in male and female “physiological environments” due to differences in hormonal regulations or growth metabolism in male and female broiler chickens [13]. The proportion of phenotypic variance that was due to maternal permanent environmental effects was low in the current study but not negligible. This might be due to the relatively older age of the birds since a maternal permanent environmental effect often influences birds at an early age and decreases as they grow older, which is in agreement with different studies in poultry [8, 9, 13, 14]. The genetic variance and heritability estimates of BW differed between the two ages and the genetic correlation of BW between the two ages was significantly different from 1. This is consistent with Mebratie et al. [8], who reported different heritability estimates for BW at three ages and genetic correlations (standard errors in parenthesis) of BW between the three ages that ranged from 0.81 (0.05) to 0.97 (0.01) in broiler chickens. Aslam et al. [14] also reported genetic correlations between BW at six ages that ranged from 0.86 (0.06) to 0.98 (0.01) in turkeys, which significantly deviated from 1. Differences in heritability estimates between the two ages and genetic correlations of BW between the two ages suggest that genes that affect BW might change with age in broiler chickens. This confirms the statement by Schaeffer [15], which highlighted that there are genes, which “turn on” and “turn off” with age of animals, causing changes in physiology and performance. Alternatively, the same genes may increase or decrease in their effect.

In general, the different heritability estimates of BW for males and females and the lower than 1 genetic correlations of BW between males and females at the two ages suggest that there is sex-by-genotype interaction for BW and that the genetic background of BW might be partly different in male and female broiler chickens. Similarly, heritability estimates of BW at the two ages and the genetic correlations of BW between the two ages suggest that there is an age-by-genotype interaction for BW and that the genetic background of BW in broiler chickens changes between the two ages. Therefore, genetic evaluation of BW in broiler chickens should take sex and age differences in to account, otherwise assuming genetic correlations of 1 between sexes and ages and average heritability estimates across sexes and ages may decrease the accuracy of selection and genetic gain. One can argue that the genetic correlations are too high to make such a conclusion, however, they are significantly different from 1, thus male and female records as well as records at the two ages cannot simply be considered as the same trait. Instead, phenotypes on male and females and at the two ages should be considered as separate traits in order to maximize the accuracy of predicted breeding values. Considering male and female records and records at the two ages as separate traits will of course cost extra computation time, but this is negligible compared to the very expensive selection experiments and the potential improvement in accuracy and genetic gain.

The heritability estimates of FI and Gain for males and females at the two ages were moderate (0.26–0.38) but lower than those reported by Aggrey et al. [16], i.e. 0.46 and 0.48 at 5 and 6 weeks of age, respectively. These authors also reported heritability estimates of 0.48 and 0.51 for Gain at 5 and 6 weeks of age in broiler chickens, respectively. However, our estimates are slightly higher than those of Begli et al. [9], who reported average heritability estimates of 0.24 for FI in chickens from weeks 2 to 10, and of Case et al. [17], who reported heritability estimates of 0.25 for FI in turkeys. Similar to the observed sex and age differences in genetic parameters for BW, the different heritability estimates for FI and Gain between males and females and the genetic correlations between them that were significantly different from 1 suggest that there is a sex-by-genotype interaction for FI and Gain. Moreover, the higher genetic correlations for FI and Gain between males and females at the earlier age (${\text{t}} - 6$) than at the later age (${\text{t}}$) suggest that there is a sex-by-age interaction for FI and Gain that increases with age, as the broilers start to attain sexual maturity.

The significantly different heritability estimates of FI and Gain in males and females and the genetic correlations between sexes, which differed significantly from 1, suggest that the genetic background of these traits might be partially different in males and females. Similarly, the significantly different heritability estimates for FI and Gain at the two ages and the genetic correlations of the traits between the two ages, which differed significantly from 1, suggest that there is an age-by-genotype interaction between these traits. Moreover, it suggests that the genetic background of FI and Gain might differ partly between the two ages. Therefore, models for the genetic analysis of the two traits should consider the sex and age in order to increase accuracy of selection and genetic gain. The moderate genetic correlation (0.30–0.50) within production traits (BW and Gain) and between BW and FI (0.49–0.63), as well as the high genetic correlation between FI and Gain (0.82–0.89) in the current study, is not surprising since higher BW and Gain might require higher feed intake and vice versa. Case et al. [17] reported a genetic correlation of 0.67 between BW and FI and a genetic correlation of 0.41 between BW and Gain in turkeys.

#### Genetic parameters for the two RFI definitions

Previous studies have reported low to moderate heritability estimates (0.10–0.49) for phenotypic RFI for male and female broiler chickens at different ages [9, 16, 18]. In pigs, Shirali et al. [7] reported low heritability estimates for ${\text{RFI}}_{\text{P}}$ (0.20 (0.03)) and ${\text{RFI}}_{\text{G}}$ (0.15 (0.03)), respectively. Our heritability estimates of ${\text{RFI}}_{\text{P}}$ (0.18–0.28) were within the range of those previously reported in chickens; although heritability estimates of ${\text{RFI}}_{\text{G}}$ are rare in the literature; our estimates (0.14–0.26) were within the range of previous estimates of ${\text{RFI}}_{\text{P}}$ in broiler chickens. In addition, the estimates of genetic variance and heritability for ${\text{RFI}}_{\text{P}}$ were higher than for ${\text{RFI}}_{\text{G}}$ (0.14–0.26), which was expected since the genetic variance of ${\text{RFI}}_{\text{P}}$ is influenced by the residual covariance between the component traits of feed intake and production traits, in contrast to the genetic variance of ${\text{RFI}}_{\text{G}}$ [3]. Estimates of heritability for ${\text{RFI}}_{\text{P}}$ and ${\text{RFI}}_{\text{G}}$ differed significantly between males and females and at the same age and between the two ages for the same sex. The significantly lower than 1 genetic correlations of the two RFI definitions between males and females at the same age, suggest that there is a sex-by-genotype interaction for these traits. Similarly, the significantly lower than 1 genetic correlations for the two RFI definitions between ${\text{t}}$ and ${\text{t}} - 6$ days at the same sex suggest that there is an age-by-genotype interaction for these traits. In agreement with our findings, Aggrey et al. [16] noted a moderate but significantly lower than 1 genetic correlation of 0.59 between phenotypic RFI of broiler chickens measured at two ages (28–35 and 35–42 days of age). In our study, estimates of the genetic correlation between ${\text{RFI}}_{\text{P}}$ and ${\text{RFI}}_{\text{G}}$ were high but significantly different from 1 at ${\text{t}}$ days of age for both sexes, however at ${\text{t}} - 6$ days of age the genetic correlations were high and not significantly different from 1. This suggests that selection for FE using either of these measures in breeding programs will yield the same genetic response at ${\text{t}} - 6$ days but may not result in the same response at ${\text{t}}$ days of age. Shirali et al. [7] conditioned FI on average daily gain and a body composition trait (lean meat percentage) and reported a genetic correlation (standard error in parentheses) of 0.92 (0.04) between ${\text{RFI}}_{\text{P}}$ and ${\text{RFI}}_{\text{G}}$ in pigs, which was high but significantly different from 1.

The non-zero estimates of the genetic correlations (0.04–0.32) between ${\text{RFI}}_{\text{P}}$ and production traits (BW and Gain) in our study were as expected based on the definition of ${\text{RFI}}_{\text{P}}$ since it is a component of FI that is phenotypically, but not genetically, independent of production traits. Genetic correlations of ${\text{RFI}}_{\text{P}}$ with production traits can vary considerably in sign and magnitude, depending on the genetic and phenotypic parameters of its component traits [3]. Moreover, the genetic covariance of ${\text{RFI}}_{\text{P}}$ with production traits involves the environmental covariance between feed intake and production traits, therefore the partial phenotypic regression coefficients of ${\text{RFI}}_{\text{P}}$ and production traits do not ensure that ${\text{RFI}}_{\text{P}}$ is genetically independent of production traits [3]. According to Kennedy et al. [3], ${\text{RFI}}_{\text{P}}$ will be genetically independent of production traits only if the heritabilities of FI and production traits are equal and the genetic correlations between them are equal to the corresponding environmental correlations. In the same context, the phenotypic correlation between ${\text{RFI}}_{\text{G}}$ and production traits is not necessarily 0, except when heritabilities of FI and production traits are equal and the genetic correlations between FI and production traits are equal to their corresponding environmental correlations [3]. Our results confirm this, since the phenotypic correlations of ${\text{RFI}}_{\text{G}}$ with production traits were low (− 0.002 to − 0.27), whereas the phenotypic correlations of ${\text{RFI}}_{\text{P}}$ with production traits were 0. Aggrey et al. [16] reported low (− 0.05 to 0.06) genetic correlations of ${\text{RFI}}_{\text{P}}$ with average daily gain and moderate genetic correlations (0.31–0.45) of ${\text{RFI}}_{\text{P}}$ with metabolic BW in broiler chickens. In pigs, Shirali et al. [7] reported a moderate genetic correlation (0.35) between ${\text{RFI}}_{\text{P}}$ and average daily gain and phenotypic correlations of − 0.30 between ${\text{RFI}}_{\text{G}}$ and average daily gain.

The estimate of the genetic variance of FI was considerably and significantly higher than the estimate for either ${\text{RFI}}_{\text{P}}$ or ${\text{RFI}}_{\text{G}}$ for males and females at the two ages, which means that most of the variation for FI is determined by production traits (BW and Gain). For example, at ${\text{t}}$ days of age, 84.3 and 82.2% of the genetic variation in FI is determined by production traits for males and females, respectively. Moreover, heritability estimates were considerably lower for ${\text{RFI}}_{\text{P}}$ and ${\text{RFI}}_{\text{G }}$ than for FI at both ages, which might be because the genetic correlations between FI and production traits were higher than the environmental correlations between the traits. Kennedy et al. [3] reported that the genetic variability in ${\text{RFI}}_{\text{P}}$ relative to feed intake depends on the phenotypic correlation between FI and production traits (BW and Gain), which is a function of the heritabilities of FI, the heritabilities of production traits as well as the genetic and environmental correlations between FI and production traits. The heritability of ${\text{RFI}}_{\text{P}}$ increases as the genetic covariance between FI and production traits decreases and as the environmental correlation between feed intake and production traits increases relative to the genetic correlation [3].

Estimates of genetic correlations between FI and ${\text{RFI}}_{\text{P}}$ ranged from 0.51 to 0.66, which is in line with the results of Kennedy et al. [3], who stated that genetic correlations between FI and ${\text{RFI}}_{\text{P}}$ are highly positive except, when the heritability of FI is low compared to that of the production traits and their genetic and environmental correlations are both high. In such situations, the genetic correlation between FI and ${\text{RFI}}_{\text{P}}$ can be even negative [3]. In our study, genetic correlations between FI and ${\text{RFI}}_{\text{P}}$ were higher than between FI and ${\text{RFI}}_{\text{G}}$ because the genetic correlation between FI and ${\text{RFI}}_{\text{P}}$ involves the environmental covariance between FI and production traits, in contrast to the genetic correlation between FI and ${\text{RFI}}_{\text{G}}$. In addition, the phenotypic correlations between FI and the two RFI definitions being lower than their corresponding genetic correlations might indicate that the environmental covariance between FI and production traits is lower than the corresponding genetic covariance. Pym and Nichols [19] noted that phenotypic correlations between traits such as BW, Gain, FI and feed conversion ratio are generally lower than their corresponding genetic correlations. Aggrey et al. [16] reported genetic correlations of 0.51 and 0.56 between FI and ${\text{RFI}}_{\text{P}}$ in broiler chickens at 35–42 and 28–35 days of age, respectively, which are in line with our estimates at ${\text{t}} - 6$ days of age.

#### Superiority of the selected group

Our results show that direct selection on one of the 12 traits included in the model results in a positive correlated response on the other traits, including a positive correlated response in FI (results not shown), which is not desirable. The observed positive correlated responses for the traits in our study is a reflection of the moderate to high positive genetic correlations between them. The Bayesian analysis suggested that direct selection for low ${\text{RFI}}_{\text{G}}$ does not result in a correlated response in production traits since the model ensures that genetic correlations between ${\text{RFI}}_{\text{G}}$ and production traits are 0. However, direct selection for low ${\text{RFI}}_{\text{P}}$ results in a correlated response in production traits since ${\text{RFI}}_{\text{P}}$ is derived using phenotypic partial regression coefficients, which ensure that phenotypic correlations between phenotypic RFI and production traits are zero but genetic correlations between the traits are not necessarily 0. The correlated response in FI to selection for low ${\text{RFI}}_{\text{G}}$ was very similar to the direct response to selection for low ${\text{RFI}}_{\text{G}}$ at the same sex and age. This is in agreement with Kennedy et al. [3], who noted that the direct response in ${\text{RFI}}_{\text{G}}$ is expected to be equal to the correlated response in FI when selection is on ${\text{RFI}}_{\text{G}} ,$ because there is no change in FI due to BW and production. However, direct genetic response for ${\text{RFI}}_{\text{P}}$ and correlated response for FI will only be equal when there is no correlated response for production traits. Otherwise, the correlated response for FI to direct selection of ${\text{RFI}}_{\text{P}}$ depends partly on the correlated response in production traits. If the correlated response for production is positive, FI will decrease less which also means a positive response for FI, which is not desirable, and vice versa.

Superiority of the selected group was slightly but not significantly lower when selection was for ${\text{RFI}}_{\text{G}}$ compared to selection for ${\text{RFI}}_{\text{P}}$ in our study. This is in agreement with Kennedy et al. [3] who reported that response to selection for low ${\text{RFI}}_{\text{G}}$ is less than or equal to response to selection for low ${\text{RFI}}_{\text{P}}$ because selection for low ${\text{RFI}}_{\text{P}}$ results in a reduction of the proportion of feed intake used for production. Moreover, Kennedy et al. [3] noted that response to selection for ${\text{RFI}}_{\text{G}}$ increases if the genetic correlation between feed intake and production traits is low or the heritability of feed intake is higher than the heritability of production traits. In our study, the heritability of FI was higher than that of production traits; however, the genetic correlation between FI and production traits was moderate to high.

Our results show that selection for FE based on ${\text{RFI}}_{\text{P}}$ or ${\text{RFI}}_{\text{G}}$ gives the same genetic response at ${\text{t}} - 6$ days but might result in a different response at ${\text{t}}$ days of age in both sexes. Since, ${\text{RFI}}_{\text{G }}$ captures the efficiency of birds in nutrient utilization irrespective of energy requirements for production and maintenance, it is easier to explain results from selection on ${\text{RFI}}_{\text{G}}$ to stakeholders than selection on FI or ${\text{RFI}}_{\text{P}}$, which are not genetically independent of production traits.

## Conclusions

Genetic parameters were estimated simultaneously for feed intake, body weight, and body weight gain using a multi-trait Bayesian analysis. The heritability estimates of the traits were moderately high but significantly different between males and females at the same age. Moreover, estimates of the genetic correlation for BW, FI and Gain between males and females at the same age were significantly different from 1, which suggests that these traits are influenced by a sex-by-genotype interaction in addition to direct genetic and maternal permanent environmental effects. Similarly, the different heritability estimates for BW, FI and Gain between the two ages within sexes and estimates of genetic correlations of BW, FI and Gain between the two ages within sexes show that there is an age-by-genotype interaction for these traits. In our study, heritability estimates for ${\text{RFI}}_{\text{P}}$ and ${\text{RFI}}_{\text{G}}$ differed significantly between sexes and between the two ages. Furthermore, estimates of the genetic correlation between the two RFI definitions were significantly different from 1 at an older age but not at a younger age. Direct selection for low ${\text{RFI}}_{\text{P}}$ resulted in a decrease in FI and a decrease in production traits, whereas direct selection for low ${\text{RFI}}_{\text{G}}$ resulted in a correlated response of decreasing FI, without a significant change in production traits.

## Data Availability

Data supporting this paper were obtained from Cobb-Vantress poultry Research Company and are available only upon agreement with Cobb-Vantress and should be requested directly from the company.
